# Why do adolescents attempt suicide? Insights from leading ideation-to-action suicide theories: a systematic review

**DOI:** 10.1038/s41398-024-02914-y

**Published:** 2024-06-27

**Authors:** Jaclyn S. Kirshenbaum, David Pagliaccio, Alma Bitran, Elisa Xu, Randy P. Auerbach

**Affiliations:** 1 Department of Psychiatry, Columbia University, New York, NY USA; 2 Division of Child and Adolescent Psychiatry, New York State Psychiatric Institute, New York, NY USA

**Keywords:** Psychiatric disorders, Depression

## Abstract

Suicide is a leading cause of death among adolescents, and recent suicide theories have sought to clarify the factors that facilitate the transition from suicide ideation to action. Specifically, the Interpersonal Theory of Suicide (IPTS), Integrated Motivational-Volitional Model (IMV), and Three Step Theory (3ST) have highlighted risk factors central to the formation of suicidal ideation and suicidal behaviors, which is necessary for suicide death. However, these models were initially developed and tested among adults, and given core socioemotional and neurodevelopmental differences in adolescents, the applicability of these models remains unclear. Directly addressing this gap in knowledge, this systematic review aimed to (1) describe the evidence of leading ideation-to-action theories (i.e., IPTS, IMV, 3ST) as they relate to suicide risk among adolescents, (2) integrate ideation-to-action theories within prevailing biological frameworks of adolescent suicide, and (3) provide recommendations for future adolescent suicide research. Overall, few studies provided a complete test of models in adolescent samples, and empirical research testing components of these theories provided mixed support. Future research would benefit from integrating neurodevelopmental and developmentally sensitive psychosocial frameworks to increase the applicability of ideation-to-action theories to adolescents. Further, utilizing real-time monitoring approaches may serve to further clarify the temporal association among risk factors and suicide.

## Introduction

Despite considerable global prevention efforts [1], suicide remains a leading cause of death among adolescents [2]. Broadly, adolescence is a developmental period characterized by substantial social change, including greater reliance on peer support [3–5], burgeoning romantic relationships [3], and enhanced autonomy [6]. For some, these changes cause stress [7, 8], which may contribute to the increased occurrence of affective disorders [9, 10] and suicide attempts [11, 12] during adolescence. Accordingly, leading suicide theories have sought to clarify how stressors and related risk factors facilitate the emergence of suicidal thoughts and behaviors (STB).

Several suicide theories have operationalized pathways from suicidal ideation to behaviors. The most widely recognized theories for examining “ideation-to-action” pathways in adolescent populations include the Interpersonal Theory of Suicide (IPTS; Joiner [13]; Van Orden et al. [14]), Integrated Motivational-Volitional Model (IMV; O’Connor [15]), and Three Step Theory (3ST; Klonsky and May, [16]). According to IPTS, the simultaneous presence of thwarted belongingness, perceived burdensomeness, and hopelessness about interpersonal states leads to intense active suicidal desire, and it is necessary for individuals to have a low fear of death and high pain tolerance to transition to suicidal behavior. The IMV is a three-part model that delineates background factors and triggering events (pre-motivational phase) that increase vulnerability for suicidal ideation. Suicidal ideation and intent form through constructs of defeat and entrapment, and transition to suicidal behavior depends on volitional moderators, which include but are not limited to capability factors outlined in IPTS. The most recently developed theory, 3ST, requires the simultaneous presence of pain and hopelessness to contribute to suicidal ideation, which intensifies only if pain overwhelms feelings of connectedness. Similar to the other theories, the capability to act on suicidal thoughts leads to suicide attempts (for similarities and differences across theories, see Table 1). Generally, these models were developed and tested among adults and later extended to adolescent populations. However, there are important interpersonal, socioemotional, and neurodevelopmental differences between youth and adults that may impact these models [17]. Therefore, the aim of this systematic review is to (1) delineate evidence for these ideation-to-action theories among adolescents, (2) incorporate aspects of these theories within recent frameworks that highlight biological components as risk factors for adolescent suicide, and (3) provide recommendations for future adolescent suicide research.

**Table 1:** Comparison of ideation-to-action theories.

Factors Leading to Suicidal Ideation	**IPTS**	**IMV**	**3ST**
	**Thwarted Belongingness**	**Vulnerability factors** (e.g., Personality, Socio-environmental, Stress)	**Pain x Hopelessness**
	**x**	↓	⇣
	**Perceived Burdensomeness**	**Defeat/Humiliation**	**Pain > Connectedness**
	**x**	**↓ x TSM** (e.g., Problem-Solving, Cognitive Biases)	⇣
	**Hopelessness**	Entrapment	
	**x**	**↓ x MM** (e.g., Thwarted Belongingness, Burdensomeness, Future Thoughts, norms)	
Factors Leading to Suicide Attempts	**Acquired Capability**	**Volitional Moderators** (e.g., Acquired Capability, Suicide Exposure, Impulsivity)	**Suicide Capability** (Acquired, Dispositional, Practical)

Key constructs are bolded.

*IPTS* Interpersonal Theory of Suicide, *IMV* Integrated Motivational-Volitional Model, *3ST* Three-Step Theory, *TSM* Threat to Self Moderators, *MM* Motivational Moderators, x Moderation, ↓ Temporal Order, ⇣ Logical Order.

## Method

PRISMA guidelines were applied to prepare this systematic review [18]. PubMed and Google Scholar searches were conducted to identify empirical articles testing the IPTS, IMV, and 3ST beginning from the dates that theories were published through January 1, 2023. The search terms included the name of the theory (e.g., “3ST”, “Three-Step Theory”) and suicide-related terms (e.g., “suicide attempt,” “suicidal ideation,” “suicidal behavior”), in combination with “adolescents” or “youth.” See Supplement for a full list of search terms. Review articles identified in our search were scanned for additional citations (*n* = 14 citations). Empirical articles were included if they: (1) were published in an English-language peer-reviewed journal, (2) included participants under 18 years old and not exceeding 21 years old, (3) tested suicidal ideation and/or suicidal behaviors as outcomes, and (4) evaluated core constructs of the IPTS, IMV, or 3ST.

**Fig. 1:**
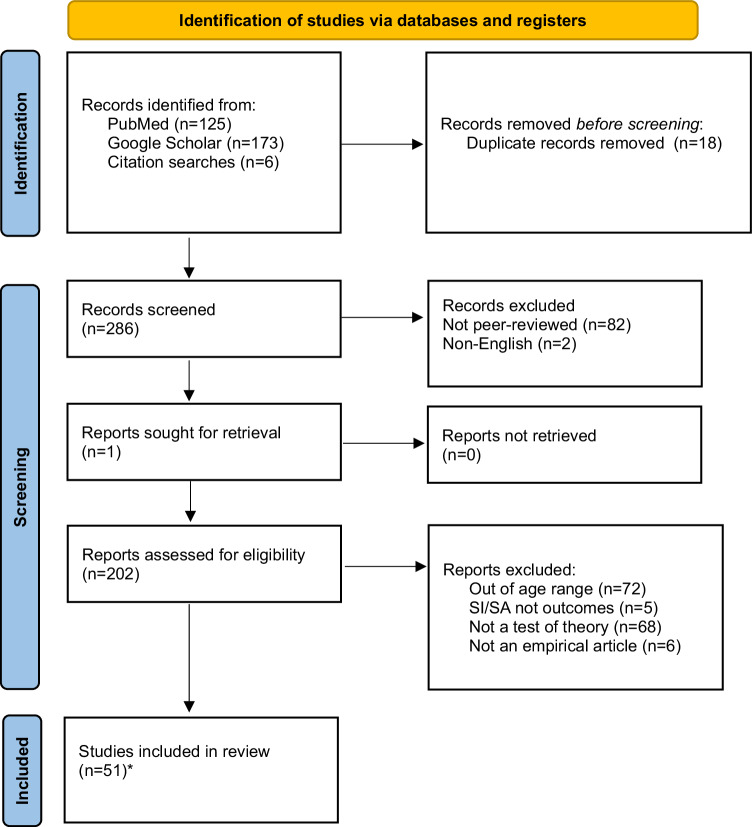
PRISMA flow diagram depicting number of identified and included articles for IPTS. *51 with Mars et al., [73] and Rooney et al., [67] (also included in IMV and 3ST totals).

**Fig. 2:**
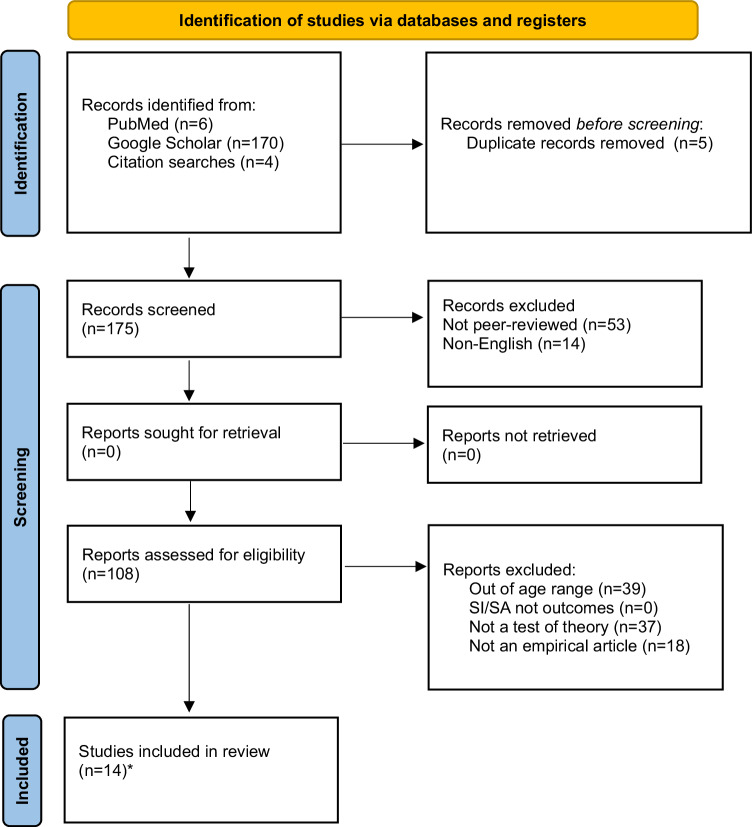
PRISMA flow diagram depicting number of identified and included articles for IMV. *14 with Mars et al., [73] and Rooney et al., [67] (also included in IPTS and 3ST totals).

**Fig. 3:**
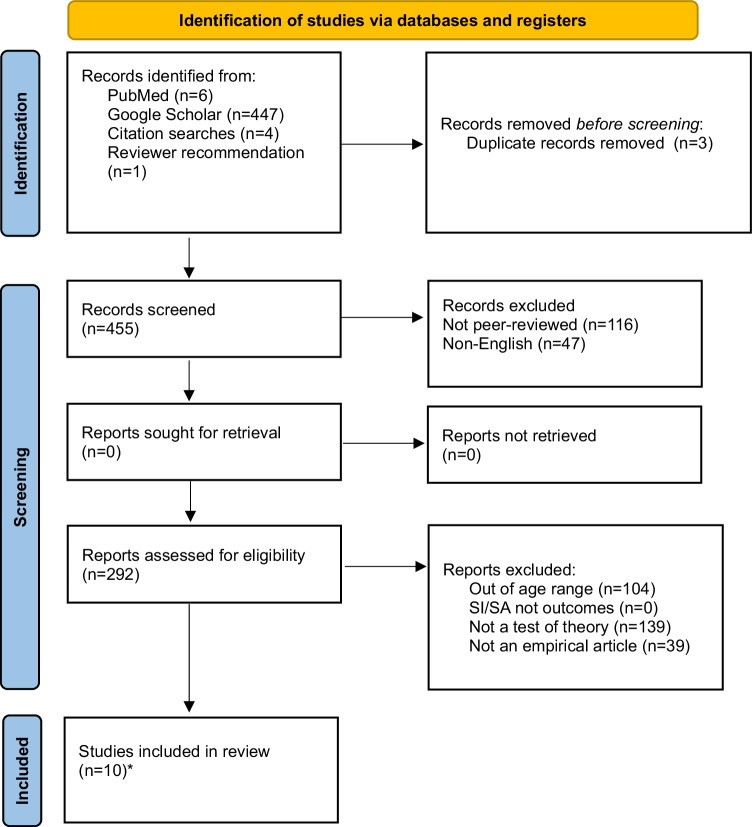
PRISMA flow diagram depicting number of identified and included articles for 3ST. *10 with Mars et al., [73] and Rooney et al., [67] (also included in IPTS and IMV totals).

After removing duplicates (*n* = 26), the systematic review yielded 916 articles. Articles were first excluded if they were not in English (*n* = 63), if they were not in a peer-reviewed journal (*n* = 251), and if they were not an empirical article (e.g., protocol paper, review, book chapter) (*n* = 63). Methods sections of articles were reviewed to ensure the age criterion was satisfied (excluded, *n* = 215), and whether suicidal ideation and/or suicidal behaviors were outcomes (excluded, *n* = 5). Article abstracts and introductions were then reviewed to identify whether the article framed the question or hypothesis within the context of the suicide theories (excluded, *n* = 244). After removing excluded articles, 75 articles were identified (IPTS, *n* = 51; IMV, *n* = 14; 3ST, *n* = 10), two of which covered all three theories. Thus, 73 unique articles were included, which encompassed research examining community (*n* = 36), clinical (*n* = 27), and at-risk samples (*n* = 13; e.g., depressed or peer-victimized youth; see Figs. 1–3 for PRISMA flow diagrams; Tables 2–4 for samples, study characteristics, and key findings).

**Table 2:** Studies testing the interpersonal theory of suicide in adolescents (*N* = 51).

Publication	Sample	*N*	Design	Assessment of suicidal thoughts and/or behaviors	Key findings
Abbott et al. [27]	Clinical	129	Longitudinal	Suicidal Ideation Questionnaire-Junior	•PB, but not TB or PB x TB, was associated with SI
Al-Dajani and Czyz [24]	Clinical	78	Four-week EMA study (post-discharge)	Daily surveys: Presence and frequency of SI. If yes, follow up item asked regarding intensity of suicidal urge.	•Daily PB x TB (family and peer) predicted same day SI •Baseline PB and previous-day PB associated with next-day SI •Neither previous-day peer TB nor family TB were associated with next-day SI
Austin et al. [131]	Community	372	Cross-sectional	SI single item: “In the past 6 months I thought about killing myself or committing suicide.” SA single item: “Have you ever tried to commit suicide?“	•Interpersonal microaggressions associated with lifetime SA •School belonging, family support, peer support, internalized self-stigma, interpersonal microaggressions, environmental microaggressions not associated with lifetime SA •School belonging, emotional neglect and internalized self-stigma associated with SI in the past 6 months
Baams et al. [45]	Community	876	Cross-sectional	SI: Positive and Negative Suicide Inventory (Negative Suicide Ideation Subscale)	•PB was associated with SI •Neither TB nor the PB x TB interaction were associated with SI
Balzen et al. [34]	Clinical	322	Longitudinal	Concise Health Risk Tracking-Self Report Scale	•Change in SI was associated with change in PB, but not TB
Barzilay et al. [40]	Community	1196	Cross-sectional	Paykel Suicide Scale	•Parental TB x PB associated with SI •Peer TB x PB not related to SI •AC associated with history of SA •AC x SI not associated with history of SA
Barzilay et al. [47]	Community	8972	Longitudinal	SI single item: “During the past 2 weeks, have you reached the point where you seriously considered taking your life, or perhaps made plans for how you would go about doing it?” SA single item: “Have you ever made an attempt to take your own life?“	•Parental, not peer, TB predicted SI at 12-month follow-up •PB not associated with SI at 12-month follow-up •SI x AC predicted SA at 12-month follow-up
Buitron et al. [28]	Clinical	180	Cross-sectional	Modified Scale for Suicide Ideation	•PB but not TB was associated with SI •PB x TB not tested
Calear et al. [41]	Community	1382	Cross-sectional	SI: Suicidal Ideation Attributes Scale (Past Month) SA single item: “How many times have you actually tried to kill yourself?” (Past Year)	•PB x TB associated with past month SI, where PB shows stronger effect •PB x TB x AC-FAD not associated with past year SA
Cero and Sifers [56]	Community	200	Cross-sectional	SA subscale of the Profiles of Student Life: Attitudes and Behavior	•Parental support (proxy for TB) reduced SA (through self-esteem, a proxy for TB and PB); Stronger for girls compared to boys
Czyz et al. [52]	Clinical	376	Longitudinal	SA single item: Diagnostic Interview Schedule for Children (DISC–IV): “Have you ever, in your whole life, tried to kill yourself or made a suicide attempt?”	•Neither familial nor peer belongingness predicted SA at 3- or 12-month time points •AC predicted SA over 12 months •PB x TB x AC did not predict SA at 3- or 12-month time points •PB x AC predicted SA at 3-month follow-up in boys, but not girls
Czyz et al. [25]	Clinical	34	Longitudinal	SI single item: At any point in the last 24 hr, did you have any thoughts of killing yourself? If yes, follow up items asked regarding the frequency and duration.	•All concurrent two-way interactions (PB x TB, TB x H, PB x H) were associated with SI frequency, duration, and urge severity •Prospectively and adjusting for previous day SI, only PB x TB and TB x H were significantly associated with next-day SI frequency and duration
Eaddy et al. [29]	Clinical	151	Cross-sectional	•Concise Health Risk Tracking Self-Report	•PB x TB not associated with SI •PB, not TB associated with SI •AC-FAD associated with SI
Elledge et al. [63]	Clinical	294	Cross-sectional	Columbia-Suicide Severity Rating Scale	•H (Lack of optimism, but not pessimism) x PB was associated with more active SI compared to passive SI
Eugene et al. [58]	At-risk (children from disadvantaged backgrounds)	2826	Cross-sectional	SI: Parent reporting on past 6 months, “Child talks about killing self” (Yes/No)	•School connectedness was not associated with SI
Grossman et al. [125]	Community	129	Cross-sectional	Self-Harm Behavior Questionnaire	•PB, not TB, associated with SI •PB x TB not related to SI •Direct measure of AC associated with SA •Exposure to painful, provocative events (a proxy for AC) associated with SA
Gulbas et al. [80]	Clinical	60	Cross-sectional	In-depth open-ended interviews	•PB, TB, and AC were endorsed by 20/30 adolescents who had attempted suicide •In many cases, PB was coded not as perceiving one’s life circumstances to be a burden to oneself
Hielscher et al. [74]	Community	216	Longitudinal	Self-Harm Behavior Questionnaire (SHBQ).	•PB x TB x AC did not predict SA during follow-up.
Hill et al. [43]	At-risk (high perceived burdensomeness)	80	Longitudinal	Beck Scale for Suicide Ideation	•PB, but not TB, associated with SI •PB x TB not tested
Hill et al. [22]	At-risk (bereaved adolescents)	58	Cross-sectional	Suicidal Ideation Questionnaire‐Junior	•PB x TB associated with SI
Hill et al. (2019)	Clinical	387	Cross-sectional	Modified Scale for Suicide Ideation	•PB, but not TB, associated with SI
Horton et al. [31]	Clinical	147	Cross-sectional	Quick Inventory of Depressive Symptomatology – Adolescent Version Self-Report	•PB, not TB, distinguished adolescents with and without SI •AC associated with SI severity
Hunt et al. [39]	Clinical	120	Cross-sectional	Suicide Ideation Questionnaire-Junior	•PB, but not TB or PB x TB, was associated with SI
Kang et al. [44]	Community	1074	Cross-sectional	Suicidal Behaviors Questionnaire	•PB, not TB, explained the association between depression and SI (and SA) •NSSI (proxy for AC) associated with SA (and SI)
Kim et al. [65]	Community	440	Cross-sectional	Korean version of the Beck Scale for Suicidal Ideation	•PB, not TB associated with SI •TB and PB associated with H, and H associated with SI
King et al. [35]	Clinical	144	Longitudinal	The Concise Health Risk Tracking Self-Report	•Measured at two timepoints, change in PB, TB, and their interaction was associated with change in SI •Change in AC not associated with change in SI •When included in the same model, PB contributed more variance to change in SI than TB
King et al. [33]	Clinical	724	Longitudinal	Self-Injurious Thoughts and Behaviors Interview Self-Report	•Greatest SA frequency occurred at highest levels of PB and NSSI (proxy for AC) as well as lowest levels of TB •TB x PB x NSSI associated with SA prior to hospitalization, but not future rehospitalization
Klonsky et al. [16] (Studies 1 and 2)	Sample 1: Clinical Sample 2: Community	S1 = 139 S2 = 426	Cross-sectional	SI single item from the Youth Risk Behavior Survey, “Have you ever seriously thought about killing yourself?” SA single item from the Youth Risk Behavior Survey, “Have you ever tried to kill yourself?”	•Across samples, AC (number of NSSI episodes) associated with lifetime SA
Li and Kwok [23]	Community	1615	Longitudinal	Child-Adolescent Suicidal Potential Index	•PB and hopelessness, but not TB, were associated with suicide potential
Liu et al. [70]	At-risk (depressed youth)	2095	Cross-sectional	Items adapted from the depression section of the National Comorbidity Survey -Replication (NCS-R) and the NCS-R-Adolescent version	•AC associated with SA, but not with SI
Mars et al. [108]	Community	4772	Cross-sectional	SI single item: “Have you ever thought of killing yourself, even if you would not really do it?” Self-harm single item: “Have you ever hurt yourself on purpose in any way (e.g., by taking an overdose of pills, or by cutting yourself)?” If responded “yes” to self-harm, SA was coded positively if: (a) they selected the response option ‘I wanted to die’ or (b) responded positively to, ‘On any of the occasions when you have hurt yourself on purpose, have you ever seriously wanted to kill yourself?’	•AC distinguished SA from SI
Matney et al. [68]	Clinical	134	Cross-sectional	Columbia-Suicide Severity Rating Scale	•Frequency and number of methods used to engage in NSSI independently associated with AC-FAD •Frequency and number of methods used to engage in NSSI independently associated with history of SA; however, AC-FAD was not associated with SAs when controlling for NSSI frequency (NSSI frequency remained significant)
Mbroh et al. [37]	Clinical	289	Cross-sectional	Columbia-Suicide Severity Rating Scale	•PB x TB did not associate with SI •NSSI and AC-FAD associated with number of SAs •AC-PT did not associate with number of SAs
Meng et al. [46]	At-risk (low-income and low parental education)	497	Longitudinal	Columbia-Suicide Severity Rating Scale	•TB at Time 1 and Time 2 associated with greater SI and SA at Time 2 and Time 3, respectively •PB at Time 1 associated with Time 2 SA, but not Time 2 SI •TB at Time 2 mediated the effect of cybervictimization at Time 1 on SI, but not SA, at Time 3 •PB at Time 2 did not mediate the effect of Time 1 cybervictimization on Time 3 SI/SA
Miller et al. [32]	Clinical	143	Longitudinal	Suicidal Ideation Questionnaire	•PB, but not TB, associated with SI, concurrently •PB x TB did not associate with SI, concurrently •Neither Time 1 PB, TB, nor their interaction associated with Time 2 SI •Only Time 1 SI and Time 2 depression severity were associated with Time 2 SI
Opperman et al. [23]	At-risk (peer-victimized youth)	129	Cross-sectional	Suicide Ideation Questionnaire-Junior	•PB x TB associated with SI •Family and school connectedness, but not peer connectedness, were correlated with SI
Phillip et al. [126]	Clinical	5	Case study	Qualitative interview assessing SI and IPTS constructs	•4/5 patients (all reporting SI) endorsed PB, while only 1 endorsed TB •Most patients with an SA endorsed both PB and TB
Rakoff et al. [42]	Community	5912	Cross-sectional	SI was measured by the response to the question (Yes/No): “In the last 12 months, did you ever seriously consider attempting suicide?”	•School connectedness (proxy for TB) not associated with prior SI •Self-esteem and worthlessness (proxies for PB) associated with prior SI
Ren et al. [69]	Community	930	Cross-sectional	Suicide Ideation and Suicide Attempt (single item): “Have you attempted suicide/had suicide ideation in the past 12 months?”	•NSSI (proxy for AC) differentiated suicide ideators from attempters •Higher pain tolerance, painful and/or provocative events, fearlessness about death, and pain sensitivity (additional proxies for AC) could not distinguish between SI and SA groups.
Rooney et al. [67]	At-risk (seriously considered suicide in past year)	821	Cross-sectional	SA: Single item assessing frequency of past-year suicide attempts	•Violence perpetration (proxy for AC) was more strongly associated with SA for youth who endorsed greater victimization, relative to those with lower victimization
Salle et al. [49]	Community	12,042	Cross-sectional	SI: “During the past 12 months, did you ever seriously consider attempting suicide?” SA: “During the past 12 months, how many times did you actually attempt suicide?”	•Sexual orientation, gender identity, and engagement in volunteering was used as a proxy variable for TB •When placed in the same model, PB and TB distinguished adolescents with vs. without SI •PB x TB not tested
Salle et al. [48]	Community	10,703	Cross-sectional	SI: “During the past 12 months, did you ever seriously consider attempting suicide?” SA: “During the past 12 months, how many times did you actually attempt suicide?”	•Sexual orientation, gender identity, presence of a caring adult, and bullying was used as a proxy variable for TB. •When placed in the same model, PB and TB distinguished adolescents with vs. without SI •PB x TB not tested
Sheftall et al. [54]	Clinical	236	Cross-sectional	Suicidal Behaviors Questionnaire	•Low parental, not peer, attachment (proxy for TB) associated with history of SA compared to psychiatric controls
Sommerfeld and Malek [51]	Community	103	Cross-sectional	Paykel Suicide Scale	•Peer and familial TB associated with SI •PB associated with SI •PB x TB not tested
Timmons et al. [53] (Study 2)	Community	1,482	Cross-sectional	SA assessed by asking whether participants had ever attempted death by suicide	•TB x home instability associated with greatest risk for SA
Van Wyk [36]	At-risk (adjudicated youth)	2,195	Cross-sectional	SI, SA, and NSSI: three dichotomized variables (Yes/No) for presence in past year, as coded from intake interviews	•PB, but not TB or PB x TB, was associated with past-year SI • AC, but not AC x PB x TB, was associated with past-year SA
Vélez-Grau and Lindsey [59]	At-risk (suicidal ideation at baseline)	210	Longitudinal	SI: “During the past 12 months, did you ever seriously think about committing suicide?” SA: “During the past 12 months, how many times did you actually attempt suicide?”	•Among those with SI, family connectedness distinguished those with vs. without SA •School (but not family) connectedness distinguished persistent SI from no SI •Self-esteem (proxy for PB) was not associated with SI/SA
Vélez-Grau et al. [72]	Community	28,442	Cross-sectional	SA single item: “During the past 12 months, how many times did you actually attempt suicide?”	•AC associated with SA
Wong and Maffini [57]	Community	959	Longitudinal	SA Single Item: Asked participants if they had attempted suicide in the past 12 months	•For most adolescents (those with lowest rate of SA), familial, school, and peer relationships predicted lower rate of future SA; However, the most consistent association across the sample was between familial connectedness and SA
Zullo et al. [38]	Clinical	151	Cross-sectional	Concise Health Risk Tracking Self-Report	•PB x TB did not associate with SI
Zullo et al. [26]	Clinical	123	Longitudinal	Concise Health Risk Tracking Self-Report	•PB, TB, and SI showed concurrent improvement following intervention.

Organized in alphabetical order.

Klonsky et al. [16] includes one clinical sample and one community sample. Mars et al. [73] and Rooney et al. [67] appear in all three ideation-to-action models.

*SI* Suicidal Ideation, *SA* Suicide Attempt, *TB* Thwarted Belongingness, *PB* Perceived Burdensomeness, *H* Hopelessness, *AC* Acquired Capability, *NSSI* Nonsuicidal Self-Injury, *FAD* Fear about Death, *PT* Pain Tolerance.

**Table 3:** Studies testing the integrated motivational-volitional model of suicide (*N* = 14).

Publication	Sample	*N*	Design	Assessment of suicidal thoughts and/or behaviors	Key findings
del Carpio et al. [87]	Community	185	Longitudinal	Self-harm single item: “Have you ever deliberately taken an overdose (e.g., of pills or other medication) or tried to harm yourself in some other way (such as cut yourself)?”	•Exposure to family self-harm, but not peer self-harm or family or peer death differentiated ideators from enactors
Glenn et al. [93]	Clinical	48	Longitudinal	Columbia-Suicide Severity Rating Scale Momentary suicidal thoughts, desire, and intent were assessed using EMA prompts	•Interpersonal negative life events (NLEs; proxy for familial and peer defeat) associated with next-day SI •Familial TB mediated association between NLEs and next-day SI •Family but not peer thwarted belongingness (proxy for defeat) mediated the relationship between NLEs and next-day suicidal thinking
Hewitt et al. [81]	Clinical	55	Cross-sectional	SI: Suicidal Ideation Questionnaire Suicide Potential: Child-Adolescent Suicide Potential Index Prior SA Single Item: “Have you ever attempted to kill yourself?”	•Controlling for depression and hopelessness, socially prescribed perfectionism, a pre-motivational factor, was associated with concurrent suicide potential •Perfectionism x life stress (conceptualized as “daily hassles”), two pre-motivational factors, associated with concurrent suicide potential (controlling for depression, hopelessness, and prior SA)
Kirtley et al. [90]	Community	743	Longitudinal (ESM)	Past-week SI: single item from the Dutch version of the SCL-90-R administered at baseline, “How often in the past week including today have you been troubled by thoughts of ending your life?”	•Past-week SI (assessed at baseline) was negatively associated with positive future thinking, a motivational moderator, (as assessed via ESM for 6 days)
Li et al. [88]	Community	1239	Cross-sectional	SI single item: “Have you thought about ending your own life in the past year?” SA single item: “Have you attempted suicide in the past year?”	•Defeat was associated with entrapment •Entrapment associated with SI •Entrapment explains the association between defeat and SI •Defeat x TSM (rumination) not associated with SI •Entrapment x MM (resilience, TB, PB) associated with SI •SI x VM (FAD, PT) not associated with SA
Mackin et al. [92]	Community	550	Longitudinal	Suicidality (SI + NSSI): Inventory of Depression and Anxiety Symptoms	• Parental support (motivational moderator) protected against future suicidality (controlling for baseline suicidality) •Social (particularly parental) support buffered the effects of interpersonal life stress, a pre-motivational factor, on suicidality at T2
Mars et al. [108]	Community	4772	Cross-sectional	SI single item: “Have you ever thought of killing yourself, even if you would not really do it?” Self-harm single item: “Have you ever hurt yourself on purpose in any way (e.g., by taking an overdose of pills, or by cutting yourself)?” If responded “yes” to self-harm, SA was coded positively if (a) they selected the response option ‘I wanted to die’ or (b) responded positively to, ‘On any of the occasions when you have hurt yourself on purpose, have you ever seriously wanted to kill yourself?’	•VM distinguished SA from SI
O’Connor et al. [85]	Community	5604	Cross-sectional	SI single item: “Have you ever seriously thought about taking an overdose or trying to harm yourself but not actually done so?” Self-harm single item: “Have you ever deliberately taken an overdose (e.g., of pills or other medication) or tried to harm yourself in some other way (such as cut yourself)?”	•Ideators and enactors differed from controls (i.e., no self-harm history) on pre-motivational and motivational factors; however, ideators and enactors did not differ from each other on these factors •Volitional factors differentiated self-harm enactors from ideators
Pollak et al. [86]	Community	74	Longitudinal	Suicide Ideation Questionnaire Self-Injurious Thoughts and Behaviors Interview-Revised	•Defeat/entrapment associated with SI history but not SA history at baseline, controlling for depressive symptoms •Defeat/entrapment predicted future SI, but did not hold controlling for depressive symptoms •Association of defeat/entrapment with SI was strongest in adolescents with greater positive future thinking
Rooney et al. [67]	At-risk (seriously considered suicide in past year)	821	Cross-sectional	SA: Single item assessing frequency of past-year suicide attempts	•Violence perpetration (proxy for AC) was more strongly associated with SA for youth who endorsed greater victimization, relative to those with lower victimization
Russell et al. [83]	Community	1045	Cross-sectional	SI single item: “Have you ever thought about ending your own life?“	•Pre-motivational factors (insomnia and nightmares) associated with defeat and entrapment •Pre-motivational factors (insomnia and nightmares) associated with SI history •Association between pre-motivational factors (insomnia and nightmares) and SI history was mediated by perceptions of defeat and entrapment
Russell et al. [84]	Community	573	Longitudinal	Self-harm thoughts single item: “Have you ever thought about taking an overdose or trying to harm yourself, but not actually done so?”	•Pre-motivational factors (mental well-being) associated with reduced likelihood of future feelings of defeat and entrapment •Association between pre-motivation (mental well-being) and self-harm ideation history was mediated by perceptions of defeat and entrapment
Yang et al. [89]	Community	4515	Cross-sectional	SI: Depressive Symptom Index-Suicidality Subscale	•Worse family functioning was associated with SI •Defeat partially mediated the relationship between family functioning and SI
Zhou et al. [82]	Community	1055	Longitudinal	SI: Beck Scale for Suicide Ideation-Screen	•Pre-motivational factors (cyber-victimization) were associated with future SI

Organized in alphabetical order.

Mars et al. [73] and Rooney et al. [67] appear in all three ideation-to-action tests and are counted towards the total count for this model.

*SI* Suicidal Ideation, *SA* Suicide Attempt, *TSM* Threat to self-moderators, *MM* Motivational Moderators, *VM* Volitional Moderators, *TB* Thwarted Belongingness, *PB* Perceived Burdensomeness, *FAD* Fear about Death, *PT* Pain Tolerance, *EMA* Ecological Momentary Assessment.

**Table 4:** Studies testing the three-step theory of suicide (*N* = 10).

Publication	Sample	*N*	Design	Assessment of suicidal thoughts and/or behaviors	Key findings
Arango et al. [106]	At-risk (peer-victimized youth)	142	Longitudinal	Suicide Ideation Questionnaire-Junior	•Family, school, and community connectedness associated with lower SI
Czyz et al. [103]	Clinical	78	Longitudinal (ESM)	*SI single item (daily survey):* “How many times did you have thoughts of killing yourself?” 0 (not at all) to 4 (all the time).	•Psychological pain and connectedness were not associated with next-day SI. •Lower (as opposed to higher) hopelessness was associated with next-day SI
Czyz et al. [104]	Clinical	32	Longitudinal (ESM)	*Suicidal Crisis:* binary variable indicating past month SA, ED visit, or re-hospitalization	•The most accurate model for predicting suicidal crisis during follow-up included variance in hopelessness, but not psychological pain or connectedness
Gunn et al. [105]	Community	4834	Longitudinal	*SI single item:* *“During the past 12 months, did you ever seriously think about committing suicide?”* *SA single item:* “During the past 12 months, how many times did you actually attempt suicide?“	•Social and parental connectedness protected against SI •In youth with SI, school connectedness protected against SA
Li et al. [102]	Community	2,259	Longitudinal	*SI single item:* “Have you had suicidal ideation in the past 6 months?” from the Suicidal Behaviors Questionnaire-Revised	•Psychological pain predicted future SI at 6-month intervals
Mars et al. [108]	At-risk (suicidal thoughts or NSSI at baseline)	690	Longitudinal	*SA Single Item:* Endorsement of “I wanted to die” when asked, “On any of the occasions when you have hurt yourself on purpose, have you ever seriously wanted to kill yourself?”	•Among those with SI at baseline, SC (defined as cannabis use, other illicit drug use, NSSI, and higher levels of intellect/openness) predicted SA during follow-up
Mars et al. [73]	Community	4772	Cross-sectional	•*SI single item:* *“Have you ever thought of killing yourself, even if you would not really do it?”* *•Self-harm single item:* “Have you ever hurt yourself on purpose in any way (e.g. by taking an overdose of pills, or by cutting yourself)?” •If responded “yes” to self-harm, SA was coded positively if (a) they selected the response option ‘I wanted to die’ or (b) responded positively to, ‘On any of the occasions when you have hurt yourself on purpose, have you ever seriously wanted to kill yourself?’	•SC (defined as exposure to self-harm) distinguished SA from SI
May et al. [101]	Clinical	52	Cross-sectional	SA: C-SSRS	•Psychological pain and hopelessness were among the most strongly endorsed motivations for SA •Pain x hopelessness not tested
Okado et al. [107]	Community	8113	Cross-sectional	*SI single item:* adolescents were asked to indicate whether they had seriously considered suicide (yes or no) or made plans to attempt suicide (yes or no). *SA single item:* adolescents indicated how many times they had attempted suicide (0 through 6 or more times).	•Social victimization (proxy for low connectedness) was associated with SI •SC (defined as behavioral disinhibition) differentiated ideators from attempters
Rooney et al. [67]	At-risk (seriously considered suicide in past year)	821	Cross-sectional	*SA single item:* assessed frequency of past-year SA	•In youth who endorsed SI, violent victimization, as well as violent victimization x violence perpetration, was associated with past-year SA

Organized in alphabetical order by first author. Mars et al. [73] and Rooney et al. [67] appear in all three ideation-to-action tests and are counted towards the total count for this model.

*SI* Suicidal Ideation, *SA* Suicide Attempt, *SC* Suicide Capability, *NSSI* Nonsuicidal Self-Injury.

### Terminology

Throughout the review, we use the term, *psychiatric controls*, to characterize individuals with psychiatric disorders but no lifetime history of suicidal ideation or attempts, *ideators* to describe individuals with current suicidal ideation and no history of attempts, and *attempters* to reflect individuals with a history of suicide attempt(s). Although not reflecting the heterogeneity of experience, these terms help improve readability and best reflect the typical groups examined in analyses. Additionally, to describe article sampling strategies, the term, *community* is used to describe participants sampled from the community, *clinical*, to describe participants recruited from inpatient and/or outpatient centers, and *at-risk* to characterize participants who met a current or lifetime threshold for suicidal ideation or other psychiatric illness or participants from disadvantaged backgrounds.

Several terms have been used differently across theories. When possible, use of the terms was consistent with each theory. For example, IPTS distinguishes passive suicidal ideation (e.g., I wish I was dead) from active suicidal ideation (e.g., I want to kill myself) and intent (e.g., suicidal plans, preparatory behaviors), whereas lethal and non-lethal attempts are considered suicidal behaviors [14]. The IMV framework does not distinguish passive from active ideation; however, suicide intent is characterized by a motivation to carry out a suicidal behavior. Suicidal behavior includes suicide attempts and deaths [19]. Similar to IPTS, the 3ST characterizes the severity of suicidal ideation, where moderate ideation is similar to passive suicidal ideation (e.g., I might be better off dead) and strong ideation is akin to active suicidal ideation (e.g., I would kill myself if I had the chance) [16]. Additionally, the 3ST defines action as suicide attempts but does not differentiate across the lethality of these attempts.

## Results

### Interpersonal theory of suicide (IPTS)

The IPTS encompasses four core constructs–thwarted belongingness, perceived burdensomeness, hopelessness about interpersonal constructs, and acquired capability–hypothesized to potentiate risk for STB. Thwarted belongingness reflects feelings of loneliness, disconnectedness, or a lack of interpersonal support (e.g., social withdrawal, family conflict). Perceived burdensomeness is characterized by feeling expendable or that one’s life is a liability to others [14]. Feelings of thwarted belongingness and perceived burdensomeness are hypothesized to lead to passive suicidal ideation (e.g., I would be better off dead). Coupled with hopelessness about thwarted belongingness and perceived burdensomeness, this can result in a more active desire to die (e.g., I want to kill myself).

Active suicidal ideation is considered necessary but not sufficient for a suicide attempt to occur. Within the IPTS, an acquired capability for suicide—reduced fear of death and higher tolerance for physical pain—is also necessary for a suicide attempt. The IPTS stresses that all four risk factors are generally experienced at their most extreme levels prior to a suicide attempt. Joiner and colleagues (2021) recently noted that an important next step for the IPTS would be to provide a comprehensive evaluation—wherein the entire model is tested rather than evaluating core components in isolation. No comprehensive test has been conducted in adolescents [20, 21].

#### Thwarted belongingness and perceived burdensomeness in relation to suicidal ideation

Within the IPTS, thwarted belongingness and perceived burdensomeness contribute independent risk for suicidal ideation as well as compounding effects when both are experienced. Yet, evidence among community and high-risk clinical samples of adolescents is mixed (Table 2). The most complete support for these IPTS predictions was observed among at-risk youth (e.g., history of peer victimization, recent loss of loved one); those who endorsed both elevated perceived burdensomeness and thwarted belongingness exhibited the highest severity of suicidal ideation [22, 23]. Similarly, among hospitalized adolescents, low connectedness in combination with high burdensomeness related to same-day [24] and next-day [25] suicidal ideation. Notably, perceived burdensomeness, thwarted belongingness, and suicidal ideation improved following a treatment specifically targeting perceived burdensomeness among inpatient youth, which revealed there are likely overlapping aspects of these interpersonal constructs [26]. Yet in other work, contrary to IPTS predictions, elevated perceived burdensomeness, but not thwarted belongingness, associated with more severe suicidal ideation [27–32], more frequent suicidal ideation (King et al. [33]), and change in suicidal ideation among clinical (Balzen et al. [34]; King et al. [35]) and at-risk youth [36]. Additionally, three studies found no interaction between perceived burdensomeness and thwarted belongingness in relation to suicidal ideation among inpatient adolescents [37, 38] or depressed adolescents [39].

Within unselected community samples, evidence also is mixed. In support of IPTS, a community sample of adolescents showed the highest severity of suicidal ideation if they endorsed both elevated perceived burdensomeness and thwarted belongingness [40]. Consistent with findings in clinical samples, perceived burdensomeness showed a stronger positive association with prior suicidal ideation [41, 42] and future suicidal ideation severity relative to thwarted belongingness [43]. In line with these findings, a mediation analysis indicated that perceived burdensomeness, but not thwarted belongingness, explained the association between depression and suicidal ideation [44]. However, another study found no interaction effects of perceived burdensomeness and thwarted belongingness in relation to suicidal ideation [45]. By contrast, other community samples found thwarted belongingness, but not perceived burdensomeness, to be associated with future suicidal ideation severity [46, 47]. Yet, a large sample of early and middle adolescents showed both perceived burdensomeness and thwarted belongingness distinguished those with and without suicidal ideation [48, 49].

Overall, there is evidence that both thwarted belongingness and perceived burdensomeness are associated with suicidal ideation. However, perceived burdensomeness is more consistently associated with suicidal ideation than thwarted belongingness in clinical samples, and findings remain mixed in community samples. This may, in part, reflect the higher prevalence and severity of both perceived burdensomeness and suicidal ideation within clinical compared to community samples. Alternatively, thwarted belongingness may be more common among clinical samples, perhaps resulting in limited variability for statistical testing.

#### Family versus peer belongingness

Thwarted belongingness is a multidimensional and complex construct. In adults, this is generally defined as a combination of loneliness and lack of reciprocal, caring connections to others. From a developmental perspective, it is important to clarify the differential impact of parents and peers on adolescent belongingness [50]. Although both low peer and familial connectedness related to high ideation in youth with high perfectionism [51], other work in a clinical sample showed neither familial nor peer belongingness associated with future suicide attempts [52] (Table 2). Other studies have shown a more consistent association between parental belongingness and suicide risk, relative to peer belongingness [40, 47]. For example, thwarted belongingness only associated with suicide attempts among adolescents when there was high instability (e.g., divorce, abandonment) in the home environment [53]. Similarly, among adolescent inpatients, attempters exhibited lower parental attachment but no difference in peer attachment, compared to psychiatric controls [54]. Moreover, among adolescents with frequent experiences of bully victimization and perpetration, low family and school belongingness (i.e., feeling like you are not part of your school) were associated with suicidal ideation, whereas peer belongingness was not [23]. There also is evidence that although peer, caregiver, and school connectedness did not relate to future suicidal ideation, suicidal ideation did associate with lower future peer and caregiver connectedness [55].

Notably, strong adolescent-parent relationships are protective against suicide risk, as enhanced parental support can reduce the likelihood of suicide attempts [56]. Among adolescent ideators, strong familial relationships, compared to school and peer relationships, most consistently associated with a lower risk for future suicide attempts [57]. Together, these findings suggest that feelings of thwarted belongingness with family during adolescence play a key role in potentiating suicide risk; however, more research related to peer belongingness is needed. It also is important to examine family and peer belongingness as complementary processes, especially as there are likely different expectations for familial support compared to peer support from early to late adolescence. Additionally, school connectedness is an understudied aspect of thwarted belongingness with mixed findings in relation to suicidal ideation [58, 59]. Understanding the ways in which an adolescent feels like a burden also may help improve social connectedness. For instance, a qualitative study that recruited adolescents based on elevated feelings of perceived burdensomeness, highlighted perceptions of being a drain on resources (e.g., time, finances) or a failure/disappointment to others. Less endorsed descriptions included relationship conflict, their own emotional distress or behavior, academic problems, and lack of belongingness [60].

#### Hopelessness

Although the IPTS was initially conceived as a three-construct theory (e.g., Joiner et al. [61]; Van Orden et al. [62]), Joiner and colleagues later revised this theory to include hopelessness about interpersonal constructs as a core IPTS construct that can lead from passive to active suicidal ideation [14]. However, few studies have tested the role of hopelessness in the context of IPTS in adolescents (Table 2). Additionally, studies of adolescents including hopelessness have tested hopelessness in general rather than hopelessness in relation to interpersonal distress. Among hospitalized adolescents, hopelessness strengthened the associations of thwarted belongingness and perceived burdensomeness with suicidal ideation [25]. Combined with high perceived burdensomeness, but not thwarted belongingness, hopelessness (lack of optimism) related to more active suicidal ideation compared to passive suicidal ideation during hospitalization [63]. Although other studies have not parsed passive and active suicidal ideation, a community sample of adolescents similarly showed that perceived burdensomeness and hopelessness were the most important factors among other variables (e.g., thwarted belongingness, resilience) contributing to suicide risk [64]. Furthermore, a community sample of high-school students showed that thwarted belongingness and perceived burdensomeness were related with hopelessness, which in turn, associated with suicidal ideation [65]. Together these findings point to hopelessness as an important contributor to suicidal ideation.

#### Acquired capability in relation to suicide behaviors

The IPTS hypothesizes pain tolerance and fear of death as key factors comprising acquired capability for suicide. Yet, research often operationalizes acquired capability based on a variety of proxy variables, including a family history of suicide, non-suicidal self-injury (NSSI), repeated suicide attempts, substance use, risky behavior engagement, or exposure to violence. Although characterized in different ways, some of the factors that contribute to pain tolerance and lowered fear of death are more interpersonal in nature (e.g., substance use and risky behaviors that tend to occur within social groups during adolescence, exposure to violence or self-harm) compared to other factors (e.g., NSSI, past attempts). Most studies have found that acquired capability (both interpersonal and non-interpersonal) associates with a history of suicide attempts in adolescents, with few indicating prospective associations with attempts (Table 2). Additionally, although physical pain tolerance and fear of death are related and both can arise from painful and provocative experiences [66], the latter has been associated with more active suicide desire (i.e., having plans to kill oneself) [29]. Interestingly, exposure to violence associated with recent suicide attempts [36, 67]; however, in combination with thwarted belongingness and perceived burdensomeness there was no association with suicide attempts [36]. In a clinical sample, frequency of NSSI, but not fear of death, was associated with a history of suicide attempts [68]. Partially consistent with these findings, NSSI and fear of death in a clinical sample were both associated with history of attempts, whereas pain tolerance was not [37]. However, in a test of different aspects of acquired capability (e.g., fearlessness about death, pain tolerance, pain sensitivity, and NSSI), only NSSI distinguished attempts from ideation [69].

Some studies have shown a stronger association between acquired capability and suicide attempts compared to suicidal ideation. In a test of acquired capability among depressed youth, intravenous compared to non-intravenous drug use was associated with suicide attempts but not suicidal ideation [70]. Consistent with these findings, acquired capability (i.e., characterized by NSSI, risky behavior engagement, and experience with violence) was related to lifetime history of suicide attempts [40, 44, 71, 72] and future suicide attempts [47] even after accounting for suicidal ideation and interpersonal risk factors (Czyz et al. [52]; King et al. [33]). Additionally, a large community adolescent sample showed that higher levels of acquired capability (i.e., exposure to family or friend self-harm) differentiated attempters from ideators [73]. Although there are likely unpublished studies that did not directly connect acquired capability with suicidal behaviors, to our knowledge, there is only one published finding in adolescents showing a null association, which may have been underpowered to test this hypothesis given relatively few attempts in a community sample of adolescents [41]. Together, there is evidence that both interpersonal and non-interpersonal aspects of acquired capability are associated with suicide attempts.

#### IPTS future directions

To date, no study has provided a comprehensive evaluation of the four IPTS components within adolescents; however, Hielscher and colleagues [74] tested a three-way interaction among thwarted belongingness, perceived burdensomeness, and acquired capability in adolescents, finding no association with suicidal ideation or attempts. Although there is support for specific IPTS hypotheses in adolescence, important empirical gaps remain. First, the role of hopelessness has been understudied, particularly as there is some evidence that hopelessness strengthens the association between interpersonal factors and suicidal ideation. Second, directly testing different aspects of acquired capability (e.g., fear of death, pain tolerance, exposure to violence) and whether these factors are due to interpersonal circumstances may help clarify the transition from ideation to action. Last, considerable variation exists within peer and school relationships (e.g., instability of romantic relationships, conflict within or between friend groups, peer pressure, school bullying), and accordingly, thwarted belongingness may not reflect a unitary construct. This could be particularly true among those who may experience cultural, racial, or ethnic marginalization [57, 75] and/or sexual or gender marginalization [76, 77]. For example, feeling left out or socially isolated due to marginalization could increase thwarted belongingness and may diminish self-worth, increasing perceived burdensomeness. Moreover, experiencing these elements across different interpersonal contexts (e.g., school, community) may exacerbate hopelessness. Additionally, given more consistent support for perceived burdensomeness compared to thwarted belongingness in clinical samples, it is important to probe this construct further in different social contexts. Measuring thwarted belongingness and perceived burdensomeness across different relationships and diverse identities may reveal important aspects of the adolescent experience that could be targeted in suicide prevention.

It also is possible that measures of perceived burdensomeness and thwarted belongingness validated in adults are less reliable in adolescents [78]. For example, a psychometric analysis indicated a different factor structure using the Interpersonal Needs Questionnaire among depressed adolescents. In particular, two thwarted belongingness items (*I feel disconnected from other people, I often feel like an outsider in social gatherings*) loaded onto their own factor, which was conceptualized as perceived isolation, and one item (*I rarely interact with people who care about me*) did not load adequately on any factor solution [39]. Consistent with these findings, removing the same three items improved model fit in another sample of psychiatric adolescents [79]. Studies using open-ended interviews may help improve measurement development of these constructs. For example, in adolescent female attempters, every adolescent reported feeling incompetent or inept (i.e., aspects of perceived burdensomeness). Yet, rather than feeling like they were themselves a burden to others, most felt that their life circumstances were a burden [80]. Additionally, most participants who attempted suicide reported experiences of thwarted belongingness, which varied across participants including changes in social networks, feeling marginalized or alienated within peer and family networks, and feeling reluctant to engage with others [80].

### Integrated motivational-volitional (IMV) model of suicide

Similar to the IPTS, the IMV model parses core differences between ideators and enactors (i.e., those who self-harm or attempt suicide; O’Connor [15]). The IMV model decomposes the transition from ideation to action in three phases: pre-motivational, motivational, and volitional. The pre-motivational phase includes background factors that provide context for suicidal thoughts and behaviors (e.g., significant life events, interpersonal stress, health-related factors, personality traits). These pre-motivational factors increase one’s vulnerability to feelings of defeat (i.e., feeling of being overcome, vanquished, or humiliated), which marks the onset of the motivational phase—initiating the formation of suicidal ideation and intent. Within the motivational phase, defeat can lead to feelings of entrapment (i.e., having no prospect of escape), which in turn, may result in suicidal ideation or intent. Notably, hopelessness is different from entrapment, as it is marked by pessimism about the future [19]. The volitional phase is characterized by factors (e.g., acquired capability, exposure to self-harm, impulsivity) that facilitate the transition from suicidal ideation to behaviors. Though defined somewhat differently, there is overlap between IPTS and IMV constructs. For instance, perceived burdensomeness and thwarted belongingness in the IPTS are contextualized as motivational moderators within the IMV model–factors that increase the likelihood that feelings of entrapment lead to suicidal ideation.

#### Pre-motivational factors

Prior research testing pre-motivational factors in adolescents supports two central tenets of the IMV model (Table 3). First, as an extension of diathesis-stress models, both vulnerability factors and stressful life events serve as primary contributors to suicidal ideation [19]. Individuals with high levels of socially prescribed perfectionism (a vulnerability factor) show increased suicide risk, particularly when experiencing high levels of daily stressors [81]. Among middle-school students, stressful life events, particularly cyberbullying, predicted future suicidal ideation [82]. Second, pre-motivational risk factors are likely to increase sensitivity to defeat or humiliation, which often may reflect social rejection and loss [19]. Supporting this hypothesis, a cross-sectional and longitudinal study showed that pre-motivational factors (i.e., poor sleep health, reduced mental well-being) associated with feelings of defeat (i.e., perceived failed struggle, loss of social rank) [83, 84]. Additionally, within the IMV framework, ideators and enactors do not necessarily differ on these pre-motivational risk factors. A cross-sectional study showed that enactors and ideators differed from controls (i.e., no self-harming thoughts or behaviors with and without psychiatric disorders) on a wide range of pre-motivational factors, including perfectionism, brooding rumination, low self-esteem, and optimism. Yet, enactors and ideators did not differ from each other on these factors [85]. As a whole, pre-motivational factors differentiate ideators and enactors from those without self-harming thoughts or behaviors, and these factors are associated with feelings of defeat, independent of depression severity.

#### Motivational factors

According to the IMV model, entrapment temporally emerges after defeat, however, both may increase risk for suicidal ideation. Studies of community samples (Table 3) have shown that defeat (i.e., feeling defeated by life, perceived failed struggle, and loss of social rank) and entrapment are associated with a history of suicidal ideation in adolescents, controlling for depression severity [83, 84, 86]. However, other work finds no difference in entrapment between ideators and controls when controlling for depression severity [87]. Another study found that entrapment accounts for the association between defeat (i.e., loss of motivation) and suicidal ideation (Li et al. [88]), and interestingly, defeat and entrapment partially account for the association between poor family functioning and suicidal ideation (Yang et al. [89]), but given these cross-sectional designs, it is difficult to establish temporal precedence. Taken together, although there are some promising preliminary findings, it is unclear whether defeat or entrapment *precede* the onset of suicidal ideation among adolescents.

#### Moderators within the motivational phase

The IMV includes a range of moderating factors in the motivation phase that facilitate or protect against the transition to ideation. Threat-to-self moderators are hypothesized to affect the link from defeat to entrapment. These include poor coping, difficulty with social problem solving, and rumination. Motivational moderators (e.g., positive future thinking, diminished social support) are hypothesized to impact the link from entrapment to suicidal ideation.

A recent study found that rumination (i.e., hypothesized threat-to-self moderator) did not moderate the association between defeat and entrapment, but thwarted belongingness, perceived burdensomeness, and reduced resilience (i.e., motivational moderators) did moderate the association between entrapment and suicidal ideation [88]. Although positive future thinking is shown to reduce suicidal ideation [90], prior research has also found that positive future thinking characterized by less realistic events may enhance suicidal ideation risk [86]. Additionally, although social support (i.e., a motivational moderator) is a critical protective factor [91] and mitigates the increase in suicidal symptoms associated with interpersonal life stress (a pre-motivational factor) [92], there are few direct tests of motivational moderators affecting the association between core motivational factors (i.e., defeat and entrapment) and suicidal ideation. Several moderators (e.g., norms, attitudes) remain untested within the context of IMV, underscoring the importance of providing a comprehensive test in adolescents.

#### Volitional factors in relation to suicide behaviors

The volitional phase of the IMV model posits specific volitional moderators that influence the transition from ideation to action and thus, differentiate ideators from enactors. This includes acquired capacity (e.g., physical pain tolerance, fearlessness of death) as well as environmental (e.g., access to means), psychological (e.g., impulsivity) and interpersonal (e.g., suicidal contagion, exposure to suicide) factors.

Exposure to suicide has been the most frequently tested volitional factor, though the type of exposure varied across studies (Table 3). Whereas adolescent enactors report more exposure to family self-harm compared to ideators, there was no difference in exposure to friend self-harm or to family or peer death by suicide [87]. In other work among inpatients, knowing a peer who attempted suicide related to increased odds of a suicide attempt [11]. However, a community sample of adolescents showed that exposure to both self-harming friends and family increased the odds of having acted on suicidal thoughts [73]. Similarly, O’Connor and colleagues (2012) showed that enactors were more likely than ideators to have had exposure to both family and friend self-harm in addition to beliefs that their peers were self-harming. Additional interpersonal factors, such as violent victimization and violence perpetration also have been associated with greater frequency of suicide attempts among ideators [67]. Several other volitional factors with smaller effects differentiate enactors and ideators, including impulsivity, substance use, depression, anxiety, or a behavioral disorder diagnoses (Mars et al. [73]; O’Connor et al. [85]). Yet, tests of several other factors (e.g., fearlessness of death, pain tolerance) were non-significant (Li et al. [88]). Collectively, exposure to family self-harm is the most consistent difference between enactors and ideators and may indicate that certain interpersonal volitional factors are critical for the transition to suicide attempts. However, longitudinal studies are needed to test the temporal transition from ideation to attempts.

#### IMV future directions

No study has provided a comprehensive evaluation of core structure of the IMV model among adolescents. However, there is evidence to support key components within each phase of the model. The IMV emphasizes defeat and entrapment as central motivators for the development of suicidal ideation. Most studies in adolescents have not tested defeat following a specific event or entrapment longitudinally. Thus, the temporal association between defeat and entrapment is unclear. One recent study identified that interpersonal negative life events, which include instances of familial and peer defeat (e.g., feeling humiliated, criticized, ignored, or bullied) were associated with next-day increases in suicidal ideation (Glenn et al. [93]) (Table 3). Furthermore familial thwarted belongingness (i.e., a motivational moderator) mediated the association between negative life events and next-day suicidal ideation (Glenn et al. [93]). Accordingly, one way in which defeat may lead to suicidal ideation is through feeling disconnected from one’s family members. Although entrapment was not tested, it is possible that different interpersonal constructs may explain familial versus peer defeat and suicidal ideation. For example, believing that peers are commonly engaging in STB may foster the perception that suicidal thinking in reaction to social defeat and humiliation is an effective way to escape pain [11, 94, 95]. Although self-reported beliefs about accepting death have been associated with suicidal ideation severity [96], measuring implicit attitudes about death in the context of interpersonal defeat and humiliation may aid in identifying who is at risk for suicide attempts, particularly for those with a self-harming history (Dickstein et al. [97]; Glenn et al. [98]) or recent suicidal ideation (Glenn et al. [99]). Such complementary approaches to probe acceptance of death may clarify the mechanisms through which peer defeat increases risk for STB. Additionally, the flexibility of the IMV, particularly with regards to moderating factors, allows the opportunity for exploring which factors, and for whom, explain the transition from the pre-motivational phase to volitional phase.

### Three-step theory (3ST) of suicide

To date, little research has investigated the 3ST in adolescents [100]. The first step focuses on an individual’s pain, which is typically psychological and/or emotional in nature. In general, pain is necessary but not sufficient to lead to suicidal ideation. Coupled with hopelessness, suicidal ideation can emerge. The second step of the 3ST underscores the importance of connectedness, which is believed to maintain a desire to live. Connectedness commonly refers to interpersonal relationships, but also can refer to an attachment to anything that yields a sense of purpose or meaning in one’s life (e.g., school, extracurricular). A combination of pain, hopelessness, and diminished connectedness may lead to severe suicidal ideation. In particular, if psychological pain hinders connectedness, even when connectedness is high, then suicidal desire may intensify [100]. The third step of the 3ST delineates the transition from ideation to action, which depends on the capability for suicide. Klonsky et al. [100] separate the capability for suicide into three key factors—dispositional, acquired, and practical. Dispositional factors are stable over the individual’s lifetime (e.g., personality traits, familial history of psychiatric disorders). Acquired factors are obtained over time, some of which are interpersonal (e.g., history of abuse) that habituate one’s fear of pain or death. Practical factors are tangible constructs, some of which are interpersonal in nature (e.g., significant alone time in one’s home, knowledge about suicide methods given family history) or non-interpersonal (e.g., access to firearms) that increase access to lethal means.

#### Pain and hopelessness

Most studies testing the 3ST have been in young adult or adult samples. However, in inpatient adolescents who recently attempted suicide, psychological pain and hopelessness were the strongest motivations to attempt suicide [101] (Table 4). In a community sample of adolescents, psychological pain related to current and future suicidal ideation across 6-month time intervals (Li et al. [102]). However, this study did not test the interaction between psychological pain and hopelessness. Contrary to these findings, a daily diary study in psychiatrically hospitalized adolescents did not find evidence that a combination of psychological pain and hopelessness predicted next-day suicidal ideation. Rather, a combination of hopelessness, burdensomeness, and self-efficacy predicted daily suicidal ideation [103] and ideation 1-month post-discharge [104]. It is possible that pain and hopelessness reflect one’s current suicidal desire rather than predictive of future suicidal ideation, which is consistent with the 3ST [100].

#### Connectedness

Connectedness is believed to protect against suicidal ideation. A community sample showed that suicidal ideation decreased with increases in social (i.e., feeling loved, wanted, accepted) and parental connectedness (i.e., closeness to parent, perceptions of warmth, satisfaction with communication) [105]. Additionally, among ideators, greater school connectedness reduced the likelihood of a future suicide attempt [105]. Similarly, in an at-risk sample of bullied adolescents, greater family, school, and community connectedness were associated with lower suicidal ideation [106]. Interestingly, certain forms of connectedness (i.e., adult support, perceived school safety, and sports participation) did not associate with a history of suicide attempt [107]. However, among ideators, higher academic grades related to a lower suicide attempt risk [107]. This study highlights that school connectedness in the form of academic accomplishments may be particularly protective against suicide attempts. Additionally, among inpatient attempters, a greater desire to die was not associated with a lack of interpersonal influence or help-seeking [101]. As these adolescents had recently attempted, it is possible that the severity of suicidal desire reached the point at which psychological pain exceeded connectedness. However, in psychiatrically hospitalized adolescents, ideation duration, hopelessness, burdensomeness, and self-efficacy, but not family or friend connectedness or psychological pain, predicted next-day suicidal ideation [103] and ideation 1-month post-discharge [104]. Although there is limited research, most studies show that social connectedness protects against STB (Table 4). As many of these studies have not examined connectedness in combination with psychological pain or hopelessness, it is not clear whether connectedness is sufficient to reduce suicidal ideation among adolescents, or whether higher amounts of psychological pain and hopelessness increase suicidal ideation regardless of connectedness.

#### Suicide capability

There is some support for suicide capability in the context of 3ST (Table 4). In a large community sample, Mars et al. [73] found that exposure to family or friend self-harm (i.e., both suicidal and non-suicidal behavior), which is an interpersonal aspect of acquired capability (i.e., pain habituation) and practical capability (i.e., knowledge of methods), most strongly differentiated ideators from attempters. However, longitudinally, dispositional factors (i.e., intellect, openness) in addition to acquired capability factors (i.e., drug use, NSSI) were more strongly associated with the emergence of a suicide attempt relative to exposure to self-harm [108]. It is possible that factors associated with a history of an attempt differ slightly from factors that predict the emergence of attempts. Alternatively, other forms of pain habituation may be associated with suicide attempts. For example, violent victimization have been associated with a greater frequency of suicide attempts among ideators [67]. Similarly, there may be different dispositional factors that associate with a past or future suicide attempt. Whereas Mars and colleagues [108] found that intellect and openness was associated with the emergence of a future attempt, Okado and colleagues (2021) indicated that behavioral disinhibition was related to one’s attempt history [107]. Overall, suicide capability contributes to differences between ideators and attempters, where different acquired and dispositional factors may temporally relate to suicide attempts. It is important to note, however, that a study examining the temporal relationship among these factors would test the predictive utility of the 3ST, and not necessarily the validity of the theory, as described in Klonsky et al. [100].

#### 3ST future directions

Although full tests of the 3ST have been conducted in adults [16, 109, 110], there is less evidence among adolescents, particularly regarding aspects within the second and third steps of the 3ST. Within the second step, social connectedness reduced risk for suicidal ideation [105, 106]. Although connectedness most commonly reflects interpersonal factors, Klonsky & May [71] describe connectedness as broadly reflecting anything that is purposeful. For example, for adults, job security or having a job with specialized skills may be a source of connectedness [111], and among adolescents, commitment to academics or other extracurricular activities could be sources of connectedness, particularly if social ties are limited. Within the third step, acquired and dispositional aspects of suicide capacity also are not well understood. As acquired factors mainly revolve around habituation to pain, understanding one’s pain threshold over time (Glenn et al. [112]), may help to understand whether acquired capability provides explanatory power in predicting attempts. Additionally, probing interpersonal circumstances of acquired capability, such as suicide contagion, which is common in youth, or befriending others with similar risk states [113] may reveal adolescent-specific factors that facilitate the transition to attempts. For measuring dispositional factors, information about familial history of suicide and personality characteristics may elucidate the stable aspects of suicide capability. Moreover, there may be potential to develop a risk calculator to assess STB risk based on different sources of vulnerability (e.g., habituation to pain, reduced peer connectedness).

## Discussion

### Challenges and future directions

#### Current biological models of suicide

For over a decade, ideation-to-action theories of suicide have guided empirical research on adolescent suicide. Recent advancements in understanding neurodevelopment [114, 115] in combination with methodological innovations probing neural activity and the stress-response system in vivo have led to novel biological models of suicide in adolescents. The aim, herein, is to expand on current biological frameworks of suicide by discussing how biological processes in the context of stress may precipitate key constructs of ideation-to-action theories that increase vulnerability to suicide.

Guided by research on the typical stress response during adolescence, Miller & Prinstein [116] suggest a failure of the biological response to acute interpersonal stress as a potential mechanism for suicidal behaviors. Specifically, interpersonal stress in combination with distal risk factors (e.g., early life adversity) are critical to triggering the biological stress-response system. The biological stress-response system, composed of the autonomic nervous system and hypothalamic-pituitary-adrenal axis, adaptively regulates physiological reactions to stress [117]. Within existing theories, the atypical biological stress-response may contribute to inadequate coping, a key IMV threat-to-self moderator. When the amount of interpersonal stress exceeds the individual’s perceived capacity to cope with the stress and when the individual has been exposed to suicide as a way to escape stress, as is articulated in the 3ST [100], suicidal behaviors are likely to emerge [116]. Alternatively, an atypical biological stress-response system may enhance feelings of hopelessness about belongingness with others and perceived burdensomeness when encountering interpersonal stress, thus facilitating more active suicidal thoughts.

Additionally, an atypical biological stress-response system may emerge through neurodevelopmental alterations that arise during pubertal development. Expanding upon the diathesis-stress model, distal genetic and environmental factors directly influence the pubertal transition, which can potentially alter connections between brain regions that are important for ruminative processes and future-oriented thinking. In our neurodevelopmental model of STB and NSSI, we highlight key neural alterations mostly shared across STB and NSSI that increase vulnerability to self-injurious thoughts in youth [118]. When acute interpersonal stress occurs, disrupted bottom-up and top-down connections among certain subcortical and cortical brain regions may potentiate self-injurious behaviors. In the context of IPTS, IMV, and 3ST, these neural connections may increase vulnerability to STB, particularly when coupled with interpersonal stress. Overall, current biological models of suicide emphasize neural and hormonal vulnerabilities that may increase susceptibility to the downstream consequences of interpersonal stress.

#### Pubertal development

Although existing theories incorporate important aspects of suicide vulnerability (e.g., hopelessness, perceived burdensomeness, thwarted belongingness, pain, acquired capability) that are applicable across the lifespan, studies testing these models have not typically included factors that strongly shape interpersonal relationships during adolescence, most notably pubertal development. Puberty is a key biological process of adolescent development that involves brain development, which can increase sensitivity to social stress [119] and STB risk [120]. Interpersonal reactions to physical changes during puberty also are of key importance, especially in explaining gender differences in STB. For example, early maturing girls, compared to boys, tend to experience greater deviant peer affiliation, which is associated with greater levels of concurrent and future depression [121]. Thus, pubertal-related interpersonal vulnerabilities may be an early indicator of depression, and possibly, subsequent STB.

#### Sexual and gender minority youth

Physical changes during puberty also are particularly stressful for youth who identify with genders that differ from their sex assigned at birth (Clark et al. [122]). Additionally, during adolescence, youth begin to explore and disclose their sexual orientation, which for some can lead to increased risk for depression and suicide. In fact, those with a sexual orientation other than heterosexual show a more rapid increase in risk for STB before 15-years-old compared to heterosexual youth [123].

Prior research using the IPTS framework found that among adolescents and young adults with same-sex attraction, the association between sexual orientation victimization and suicidal ideation was explained by perceived burdensomeness, not thwarted belongingness [45, 124]. Consistent with these findings, transgender adolescents endorse greater perceived burdensomeness compared to thwarted belongingness [125, 126]; however in those who attempted suicide, most patients endorsed a combination of these IPTS factors [126]. In addition, perceived burdensomeness and thwarted belongingness were prospectively associated with suicidal ideation among transgender and genderqueer youth compared to cisgender youth [127]. In sexual minority youth, those who lost friends after coming out about their sexual orientation were 29 times more likely to have reported a history of suicide attempt than those who did not lose friends after disclosing their sexual orientation [128]. The loss of friends after disclosing sexual orientation and the fear of disclosing are stressful events that may, in turn, increase sensitivity to social stress, leading to increases in perceived burdensomeness [129, 130]. Interpersonal microaggressions among transgender youth [131] and everyday discrimination also have been potent variables associated with suicidal behaviors in sexual and gender minority adolescents [132], which could increase psychological pain and hopelessness. Consistent with 3ST, these factors combined with reduced connectedness from friends may intensify suicidal ideation.

Additionally, consistent with IPTS, IMV, and 3ST, acquired capability was associated with suicide attempts in transgender and gender nonconforming youth [125]. Open-ended interviews of sexual and gender minority young adults with a recent suicide attempt consistently revealed that family members’ chronic invalidation or rejection of their identities during childhood and adolescence contributed to feelings of psychic pain and fear; increasing pain habituation particularly during this developmental period may have superseded any pain and fear of harming oneself (Clark et al. [133]).

Providing greater support for sexual and gender minority youth, particularly throughout the pubertal transition, can help mitigate suicide risk. Several studies have tested promising strategies to increase empathy, reduce bias, and increase knowledge surrounding sexual minorities in young adults [134]. In schools, incorporating programs to reduce stigma based on sexual orientation (e.g., gay-straight alliances) lower the odds of peer victimization and attempt [135]. It is possible that these programs are reducing risk through lowering perceived burdensomeness. As suggested by Williams and colleagues [136], suicide prevention in sexual and gender minority youth may require multiple sources of support to reduce the effects of perceived burdensomeness and thwarted belongingness, such as school-wide training in gender-affirming language (e.g., pronouns). Additionally, school curricula with inclusive sexual education, incorporation of reading materials from LGBTQ+ authors, and parent education resources may facilitate acceptance, promote self-worth, and increase hopefulness while also reducing perceived burdensomeness and thwarted belongingness [137, 138].

#### Ethnically and racially minoritized youth

Suicide deaths among racially minoritized adolescents have been increasing at a dangerous rate. Recent data show significant increases in suicide deaths among Black youth [139], with Black adolescents attempting ~2 times more compared to their White counterparts [72]. Similarly, Hispanic adolescents, particularly females, seriously consider attempting suicide ~2 times more compared to their male non-Hispanic counterparts [140, 141]. Presently, there is insufficient suicide screening among Black [142] and Hispanic [143] youth. Leading suicide researchers focusing on racial and ethnic disparities have proposed frameworks to address this issue. By combining aspects of intersectional theory and the IPTS, Opara et al. [144] has hypothesized that individual characteristics (e.g., thwarted belongingness, perceived burdensomeness) are contextualized within one’s immediate environment (family and peer relationships), which interacts with factors from the larger community (e.g., socioeconomic status, exposure to violence, racial discrimination), culture, and society of institutional discrimination (e.g., racism, classism, stigma) to potentially increase suicide risk among Black youth. This framework highlights racial discrimination existing across all levels of an individual’s environment. Moreover, the effects of subtle forms of discrimination (i.e., microaggressions or identity-related aggressions from other students) may be particularly potent in leading to suicidal ideation in Black youth [145, 146]. Although risk factors for Hispanic youth differ, there are systemic societal factors (e.g., anti-immigrant attitudes, acculturation) that can contribute to interpersonal stress (e.g., bullying, family conflict) within one’s immediate environment, which may increase vulnerability to STB [140, 147]. The 3ST also could be particularly useful in testing the effects of racism on suicidal ideation in adolescents given the consequences of racism include diminished academic performance and reduced connectedness with others [147–150], which downstream may exacerbate feelings of hopelessness. Together, addressing racial and ethnic discrimination at different levels of one’s environment may help reduce the rising rates of suicide in Black and Hispanic youth.

#### Real-time monitoring

Although traditional methods using self-reports and interviews probe adolescents’ general vulnerability to suicide, they do not capture fine-grained, temporal patterns of social experience, including interpersonal stress and connectedness. Given that ~84% of youth use mobile devices to connect with people digitally [151] and, on average, spend up to nine hours per day using their phones [152], digital communication can provide insight into real-time social experiences. Advances in mobile sensor technology (e.g., accelerometer, global positioning system; GPS, keyboard inputs) to track behavior have been successfully used to probe social interactions [153], and in combination with ecological momentary assessment, where adolescents are probed repeatedly regarding their affective and social experiences, have promise to detect imminent suicide risk [154, 155]. In addition, social media apps can be a useful tool to track changes in social engagement behaviors (e.g., reduction in posts, changes in self-referential posts), particularly before and after a suicide-related event to establish patterns of recovery [156]. Prior work suggests that interpersonal stressors occurring via social media (e.g., feeling left out or excluded online, fights or arguments on social media) can occur frequently in the weeks leading up to adolescents’ hospitalization [157, 158]. In the context of IPTS, a longitudinal study using self-reported measures found that cybervictimization predicted thwarted belongingness, which predicted future suicidal ideation and attempts [46]. As suicide-related symptom course may be nonlinear and difficult to predict [100, 159–161], employing smartphone sensor capabilities in combination with self-report in the context of social communication and engagement with social media apps may clarify *when* risk is imminent. In addition to detecting imminent risk, we also may elucidate which aspects of social connectedness or belongingness predict STB.

## Limitations

There are several limitations to address. First, this review only included articles that explicitly tested core components of formulated theories. Thus, there may be several pieces of evidence supporting components of these theories, prior to their formulation. Second, articles were not excluded based on a minimum age requirement. Although few, there are articles that included pre-adolescents or children (ages <13 years old). There are important factors that may differentially contribute to suicide risk in children compared to adolescents [162]. An important area for future research is to identity factors that contribute to suicide risk across childhood through adolescent development. Third, several studies reviewed were underpowered to test two- and three-way interactions [163], which may affect study findings, thereby impacting conclusions drawn from this review. Last, the review highlights novel inroads for the integration of biological processes; however, there is limited research integrating these factors into extant ideation-to-action theories of suicide.

## Summary

In this systematic review, there is support for key components of ideation-to-action theories; however, the need to test comprehensive models is clear. In addition, exploring the potential impact of diverse gender, sexual, and racial groups may provide insight regarding culturally sensitive mechanisms that can be targeted in future preventative-intervention strategies. Critically, integrating digital approaches also may offer an inroad to scale tracking among high-risk patients, particularly during vulnerable periods, which ideally would improve the detection of suicide risk and reduce the needless loss of life among young people.

### Supplementary information

Supplement
