# Supplementary material for: Why do adolescents attempt suicide? Insights from leading ideation-to-action suicide theories: a systematic review
Source: Transl Psychiatry. 2024 Jun 27;14:266. doi: 10.1038/s41398-024-02914-y (PMC11211511; doi:10.1038/s41398-024-02914-y)
Supplement: Supplementary file 1 — Supplement [file 41398_2024_2914_MOESM1_ESM.docx]

**Why Do Adolescents Attempt Suicide? Insights from Leading**

**Ideation-to-Action Suicide Theories: A Systematic Review**

**Supplemental Material**

Jaclyn S. Kirshenbaum^1,2^, David Pagliaccio^1,2^, Alma Bitran^1,2^, Elisa Xu^1,2^, &

Randy P. Auerbach^1,2,3^

1. Department of Psychiatry, Columbia University, New York, NY

2. New York State Psychiatric Institute, New York, NY

3. Division of Clinical Developmental Neuroscience, Sackler Institute, New York, NY

*Corresponding Author: 1051 Riverside Drive, Pardes 2407, New York, NY 10032; Email: [rpa2009@cumc.columbia.edu](mailto:rpa2009@cumc.columbia.edu)

**Table S1. Search Terms for Interpersonal Theory of Suicide in Adolescents**

| Database | PubMed | Google Scholar |
| --- | --- | --- |
| Search Period | 1/1/2005-1/1/2023 | |
| Search Terms | (“ipts” [Title/Abstract] OR “interpersonal theory of suicide*”[Title/Abstract] OR “interpersonal-psychological theory”[Title/Abstract] OR “interpersonal theory”[Title/Abstract] OR “thwarted belongingness”[Title/Abstract] OR “perceived burdensomeness”[Title/Abstract] OR “acquired capability”[Title/Abstract]) AND (“suicide”[Title/Abstract] OR “suicide attempt”[Title/Abstract] OR “suicidal ideation”[Title/Abstract] OR “suicidal behavior”[Title/Abstract]) AND (“adolescents*”[Title/Abstract] OR “youth”[Title/Abstract] OR “child*”[Title/Abstract]) AND 2005/01/01:2023/01/01[Date - Publication] AND “english”[Language] | (“interpersonal theory of suicide” or “ipts”) and (“thwarted belongingness” or “perceived burdensomeness” or “acquired capability”) and (“suicide” or “suicide attempt” or “suicidal ideation” or “suicidal behavior”) and (“adolescents” or “youth” or “child”). |

**Table S2. Search Terms for Integrated Motivational-Volitional Model in Adolescents**

| Database | PubMed | Google Scholar |
| --- | --- | --- |
| Search Period | 1/1/2011-1/1/2023 | |
| Search Terms | (“IMV”[Title/Abstract] OR “integrated motivational volitional model*”[Title/Abstract]) AND (“suicide”[Title/Abstract] OR “suicide attempt”[Title/Abstract] OR “suicidal ideation”[Title/Abstract] OR “suicidal behavior”[Title/Abstract]) AND (“adolescents*”[Title/Abstract] OR “youth”[Title/Abstract] OR “child*”[Title/Abstract]) AND 2011/01/01:2023/01/01[Date - Publication] AND “english”[Language] | (“Integrated Motivational-Volitional Model” or “imv”) and (“suicide” or “suicide attempt” or “suicidal ideation” or “suicidal behavior”) and (“adolescents” or “youth” or “child”). |

**Table S3. Search Terms for Three-Step Theory in Adolescents**

| Database | PubMed | Google Scholar |
| --- | --- | --- |
| Search Period | 1/1/2015-1/1/2023 | |
| Search Terms | (“three step*”[Title/Abstract] OR “3ST”[Title/Abstract]) AND (“suicide”[Title/Abstract] OR “suicide attempt”[Title/Abstract] OR “suicidal ideation”[Title/Abstract] OR “suicidal behavior”[Title/Abstract]) AND (“adolescents*”[Title/Abstract] OR “youth”[Title/Abstract] OR “child*”[Title/Abstract]) AND 2015/01/01:2023/01/01[Date - Publication] AND “english”[Language] | (“Three-Step Theory”) and (“suicide” or “suicide attempt” or “suicidal ideation” or “suicidal behavior”) and (“adolescents” or “youth” or “child”) |
